# Follow-Up of Thrombin Generation after Prostate Cancer Surgery: Global Test for Increased Hypercoagulability

**DOI:** 10.1371/journal.pone.0051299

**Published:** 2012-12-07

**Authors:** Matyas Benyo, Tibor Flasko, Zsuzsanna Molnar, Adrienne Kerenyi, Zoltan Batta, Tamas Jozsa, Jolan Harsfalvi

**Affiliations:** 1 Department of Urology, Medical and Health Science Center, University of Debrecen, Hungary; 2 Department of Laboratory Medicine, Medical and Health Science Center, University of Debrecen, Hungary; 3 Clinical Research Center, Medical and Health Science Center, University of Debrecen, Hungary; 4 Department of Paediatric Surgery, Division of Urology, Astrid Lindgren Children’s Hospital, Karolinska University Hospital, Stockholm, Sweden; Gentofte University Hospital, Denmark

## Abstract

Recent studies provided evidence that evaluation of thrombin generation identifies patients at thrombotic risk. Thrombin generation has a central role in hemorrhage control and vascular occlusion and its measurement provides new metrics of these processes providing sufficient evaluation of an individual’s hemostatic competence and response to anticoagulant therapy. The objective of the study is to assess a new measure of hypercoagulability that predisposes to venous thromboembolism in the postoperative period after radical prostatectomy. Pre- (day-1) and postoperative (hour 1, day 6, month 1 and 10) blood samples of 24 patients were tested for plasma thrombin generation (peak thrombin), routine hematology and hemostasis. Patients received low molecular weight heparin for thromboprophylaxis. Peak thrombin levels were higher in patients compared to controls at baseline (p<0.001), and elevated further in the early postoperative period (p<0.001). Longer general anesthesia and high body mass index were associated with increased thrombin generation after surgery (p = 0.024 and p = 0.040). D dimer and fibrinogen levels were higher after radical prostatectomy (p = 0.001 and p<0.001). Conventional clotting tests remained within the reference range. Our study contributed to the cognition of the hypercoagulable state in cancer patients undergoing pelvic surgery and revealed the course of thrombin generation after radical prostatectomy. Whilst it is unsurprising that thrombin generation increases after tissue trauma, further evaluation of this condition during the postoperative period would lead urologists to an international and well-supported consensus regarding thromboprophylaxis in order to provide better clinical outcome. Considering the routine evaluation of procoagulant activity and extending prophylactic anticoagulant therapy accordingly may potentially prevent late thrombotic events.

## Introduction

Worldwide 52% of hospitalized patients are judged to be at risk of thrombotic events including deep venous thrombosis (DVT) or pulmonary embolism (PE), which were adjudicated as the most common cause of death after pelvic cancer surgery –like radical prostatectomy (RP)– according to a prospective observational study [1]. Most contemporary RP series report rates of thromboembolic complications ranging from 0.8% to 6.2% (with the use of various prophylactic measures), so there is no doubt that RP carries a significant risk of potentially fatal venous thromboembolism (VTE) [2]. Guidelines were composed to prevent thrombotic events, but the risk assessment and prophylaxis after radical prostatectomy is still under debate [3], [4].

Hypercoagulability identifies the imbalance of the coagulation cascade toward the procoagulant condition due to an excessive activation of coagulation enzymes without clinical signs of thrombosis. Coagulation tests are used to screen for the presence of hemostasis disorders and to monitor treatment. Hypercoagulability is frequently associated with changes in prothrombin time (PT), activated partial thromboplastin time (APTT), thrombin time (TT) and fibrinogen (FNG) levels. D dimer is another well-defined test to indicate hypercoagulation and hyperfibrinolysis. Although the specificity of D dimer for DVT or PE is poor, because of fibrin produced in cancer, inflammation, infection and necrosis too, it is still a widely used initial test [5].

Recent trials measuring thrombin generation by fluorescent substrate assay proved that this is a suitable method for estimating the risk of thrombotic events under appropriate sample handling and laboratory conditions [6], [7], [8], [9]. Elevated basal peak thrombin generation was associated with a 74% increased risk of VTE published in prospective LITE data [10]. This method measures the amount of thrombin formed upon recalcification of citrated plasma and by initiation of the cascade reaction by adding exogenous activators like human recombinant tissue factor (TF) and phospholipids [8].

In the present study, we assessed the extent of hypercoagulability by measuring the plasma thrombin generation capacity in the early and late postoperative period after radical prostatectomy, using commercially available kit. To the best of our knowledge, this study is the first to evaluate the course of the hypercoagulability in cancer patients undergoing major pelvic surgery.

## Materials and Methods

In our prospective study, 24 patients with histology proven localized prostate cancer were selected (mean age 61, range 50–70 years), and followed up after laparoscopic radical prostatectomy. Patients with any anticoagulant medication or any medical condition potentially affecting coagulation assessment, such as severe chronic cardiovascular disease, advanced hepatic or renal failure, previous thrombotic events and any other kind of malignancy or previous treatment of prostate cancer were excluded from the study. For comparison 20 male age matched controls (mean age 60, range 42–76 years) without prostate cancer and with the same exclusion criteria as for the patients were recruited at the urology outpatient service. The prostate cancer was excluded by the following inclusion criteria: total Prostate Specific Antigen (tPSA) level <1.5 ng/ml, digital examination of prostate negative. The controls didn’t undergo any surgical intervention, they were healthy volunteers of the age of the study group or men seeking for treatment of mild dysuria. Written informed consent was obtained from each participant. The local ethics committee of the University of Debrecen (Debrecen, Hungary) approved the study protocol.

Low molecular weight heparin (LMWH) administration (40 mg or 4000 IU anti-Xa/day) was started one day prior to the surgical intervention and was continued for 5 weeks after the laparoscopic procedure according to the guidelines of the American Urological Association (AUA) [4]. Patients were mobilized on the first postoperative day.

Blood was collected from the antecubital vein into the appropriate Vacutainer tubes (Beckton-Dickinson, NJ USA) 1 day prior, at 1 hour, 6 days, 1 and 10 months after radical prostatectomy. Sampling was performed at least 12 hours after LMWH administration to obtain heparin free plasma from patients under therapy. PT, APTT, TT, FNG by Clauss method and d dimer were determined by an automated coagulometer (BCS-XP, Siemens, Germany) using Innovin, Pathromtin SL (Siemens, Germany), Thrombin time and Fibrinogen reagent, (Labexpert Ltd, Debrecen, Hungary), and Innovance d dimer (Siemens, Germany), respectively. Measurement of antithrombin (AT) activity (Innovance AT, Siemens, Germany) and LMWH levels (Berichrom Heparin, Siemens) was performed by using factor Xa and its chromogenic substrate. Hematology parameters such as red blood cell (RBC), white blood cell (WBC) and platelet count (PLT) were measured using a XE-2100D hematology analyzer (Sysmex, Japan). PSA level from serum was determined on Modular Analytics E170 using Total PSA Reagent (Roche Diagnostics, Germany).

For the thrombin generation assay (TGA) citrated blood samples were centrifuged at 20°C for 15 minutes at 3200 g within one hour of venipuncture. Plasma was separated into a new tube and centrifuged again (3200 g, 10 min), aliquots of the supernatant were stored at −70°C. Analysis was performed within three weeks. Individual aliquots were defrosted in a 37°C water bath by tilting for 15 minutes, vortex mixed for 5 seconds and sampled immediately into black 96 well plates (Greiner GmbH, Germany). TGA were carried out using Technothrombin® TGA kit with fluorescent substrate (1 mM containing 15 mM CaCl_2_) and reagent C (low concentration of phospholipid micelles and 50 pM recombinant human tissue factor), according to the manufacturer’s instructions. Reagent C was diluted tenfold in the reaction. The reagents and the reader with plate were preheated to 37°C. The enzyme reaction was detected with a BIOTEK Flx800 reader. Results were evaluated with KC4 TGA software. All the TGA kit, the Reader and the software were purchased from Technoclone GmbH, Austria.

For statistical analysis the patients were divided into subgroups according to the followings: tumour stage (PT2, n = 19; PT3, n = 5) additional lymphadectomy (n = 12); narcosis time (≥230 min, n = 12 and ≥230 min, n = 12); body mass index (BMI, ≤25, n = 8 and ≥25, n = 14). Preoperative data were compared to controls and the results of the postoperative samples were compared to the preoperative ones (day-1). Statistical tests were performed using GraphPad Prism version 5.00 for Windows (GraphPad Software, San Diego CA, www.graphpad.com). P values were calculated according to the distribution of the given data series, to the results of D'Agostino-Pearson and Shapiro-Wilk normality tests and the option of paring: i.e. “preoperative results” to the “results of the controls” with unpaired t test (with Welch’s correction or Mann Whitney test); “postoperative results” to the “preoperative results” with paired t test (and Wilcoxon signed rank test). According to the Gaussian distribution we used Pearson or Spearman test during correlation analysis in relation to FNG levels, conventional clotting times and TGA parameters. P<0.05 is considered to indicate statistical significance.

## Results

Based on the preoperative PSA values (mean: 8 ng/mL) and histology of the prostate biopsy specimens, radical prostatectomy was combined with regional lymphadenectomy in 12 out of 24 cases. The mean narcosis time was 233 minutes (range 140–335). Mean blood loss was 314 mL (range 50–700) which correlates with the changes in the RBC levels. No severe intra- or postoperative complications occurred.

Histological examinations revealed prostate cancer in stages between pT2a and pT3b in one case with a single lymph node metastasis (Gleason Score 5–9). The median value of PSA was reduced to the limit of detection. Salvage irradiation therapy and 6 month hormone therapy was given to 2 patients from the 6^th^ postoperative week. No relapse of malignancy was experienced in any patient in the first ten postoperative months. Eighty-three percent (20/24) of the study population was continent, and 42% (10/24) of the patients had erection with or without medication. No thrombotic complications occurred during the observational period.

Detailed follow-up laboratory results of the 24 patients are summarized in Table 1 and Table 2.

White blood cell counts were elevated one-hour postoperatively and normalized afterwards. Platelet counts were the highest on the sixth postoperative day and lowered afterwards. Prothrombin time and TT were prolonged in the one-hour postoperative samples and then returned back to the preoperative value. Activated partial thromboplastin time was prolonged preoperatively compared to the controls and shortened after the operation. Conventional clotting tests remained in the reference range after surgical procedure. Reference ranges in our laboratory are: PT: 7.5–12.8, APTT: 26.5–37.7 and TT: 15.4–23.4 [sec]. Fibrinogen levels decreased first, then doubled and decreased almost to the preoperative value at one month. D dimer level was high at one-hour postoperatively, further elevated by the sixth day and normalized afterwards.

**Table 1:** Routine test results of patients before and following radical prostatectomy.

Parameter	Reference Range or Mean±2SD of Controls	Preoperative sample^1^	Hour 1	Day 6	Month 1	Month 10
			After Surgery^1^			
tPSA *	<4.4	8.1	8.5	1.5	0.0	0.0
[ng/mL]		6.2–11.5	6.6–14.1	1.0–2.2	0.00–0.05	0.00–0.03
RBC	4.2–5.2^2^	4.7	**4.4**	**4.3**	4.5	4.6
[T/L]		4.5–5.0	4.0–4.6	3.9–4.7	4.2–5.0	4.5–4.8
			p<0.0001	p = 0.0001	p = 0.0013	NS
WBC	4.5–10.8^2^	7.2	**13.5**	7.2	**6.0**	6.4
[G/L]		6.0–7.8	11.1–16.7	5.9–8.6	4.9–6.9	5.8–7.4
			p<0.0001	NS	p = 0.0203	NS
PLT	150–400^2^	203	199	**244**	237	228
[G/L]		173–231	156–261	213–293	185–298	176–266
			NS	p<0.0001	NS	NS
PT	8.0	8.1	**8.7**	**7.6**	8.1	7.9
[sec]	7.4–8.7^3^	7.8–8.3	8.2–9.0	7.4–8.0	7.8–8.3	7.7–8.0
			p = 0.0007	p = 0.0002	NS	NS
APTT	28.1	29.3	27.2	27.1	**27.8**	28.0
[sec]	26.7–29.5^3^	27.6–30.7	25.4–29.2	26.2–30.8	26.0–30.7	26.8–30.7
			NS	NS	p = 0.0157	NS
TT	17.3	17.9	**18.5**	**16.7**	17.9	18.9
[sec]	14.5–20.2^3^	17.0–18.5	17.8–19.5	15.8–17.6	16.9–19.0	17.9–19.1
			p = 0.0035	p = 0.0007	NS	NS
Fng	1.5–4.0^2^	3.3	**2.8**	**5.3**	**3.7**	3.1
[g/L]		2.8–4.0	2.5–3.2	4.9–5.7	3.4–4.2	2.9–3. 5
			P = 0.0302	p<0.0001	0.0204	
D dimer	<0.5^2^	0.27	**0.88**	**1.16**	0.42	0.24
[mg Feu/L]		0.24–0.47	0.53–1.46	0.86–1.32	0.21–0.91	0.20–0.45
			p = 0.0010	p = 0.0010	NS	

1 Results are given as mean and ±SD or median and 25–75 percentile values, depending on the normality of the test results. P values are also calculated according to the distribution of the given data series and the option of paring: i.e. “preoperative results” to the “results of the controls” with unpaired t test (with Welch’s correction or Mann Whitney test); “postoperation results” to the “preoperative results” with Paired t test (and Wilcoxon signed rank test). Preoperative data were compared to controls and the results of the postoperative samples were compared to the preoperative ones (day-1).

2 reference range of the method applied.

3 mean±2SD of pooled control samples (n = 20) in the period of the study.

4 ND = not determined.

Bold letters indicate significant differences.

**Table 2:** Specific test results of patients before and following radical prostatectomy.

Parameter	Values of the Control Group	Preoperative sample^1^	Hour 1	Day 6	Month 1	Month 10
			After Surgery^1^			
Peak	206	**302**	**330**	**420**	289	244
thrombin	±29	±68	±91	±85	±71	±44
[nM]		p = 0.0001	p = 0.0089	p<0.0001	NS	NS
AUC	2841	**3522**	3513	**4108**	3638	3051
[nM*min]	±375	±505	±618	±468	±574	±444
		p<0.0001	NS	p<0.0001	NS	p = 0.0209
lag phase	11.8	11.6	**10.5**	12.1	11.6	11.3
[min]	±1.80	±2.21	±2.42	±2.96	±2.61	±1.60
		NS	p<0.0001	NS	NS	NS
Peak time	16.9	16.6	**14.3**	16.0	16.1	16.0
[min]	±1.92	±2.52	±3.45	±3.61	±2.99	±1.46
		NS	p = 0.0004	NS	NS	NS
LMWH	0.1^2^	NT^3^	0.03	0.07	0.06	NT^3^
[IU/mL]			0.02–0.06	0.03–0.09	0.02–0.09	
AT	103	106	89	101	102	101
[%]	92–115	97–117	74–102	95–112	93–107	94–130

1 Results are given as mean and ±SD or median and 25–75 percentile values, depending on the normality of the test results. P values are also calculated according to the distribution of the given data series and the option of pairing. Preoperative data were compared to controls and the results of the postoperative samples were compared to the preoperative ones (day-1).

2 limit of detection.

3 No LMWH therapy.

Bold letters indicate significant differences.

Thrombin generation measured by TGA was evaluated by the peak thrombin concentration (nM), the area under the curve (AUC), the lag phase, the peak time and the velocity index (V-index; Table 2. and Figure). The reaction was triggered by 5 pM tissue factor in all the measurements. Compared to the controls, peak thrombin and AUC were elevated in the patients’ preoperative samples, while the other parameters of the thrombin generation remained unchanged. The peak thrombin levels were further elevated in the early postoperative period, reaching a maximum by the sixth day, as did AUC, and normalized by the end of the first month. Significant differences in the lag phase, peak time and velocity index were seen in the postoperative one-hour samples. None of the TGA parameters correlated with the changes in fibrinogen levels except AUC on the sixth day (p = 0.0038, Pearson correlation). No correlation between conventional clotting times and changes in the thrombin generation parameters were found, except for PT and TT on the sixth day, where the correlation with the lag phase was significant.

**Figure 1:**
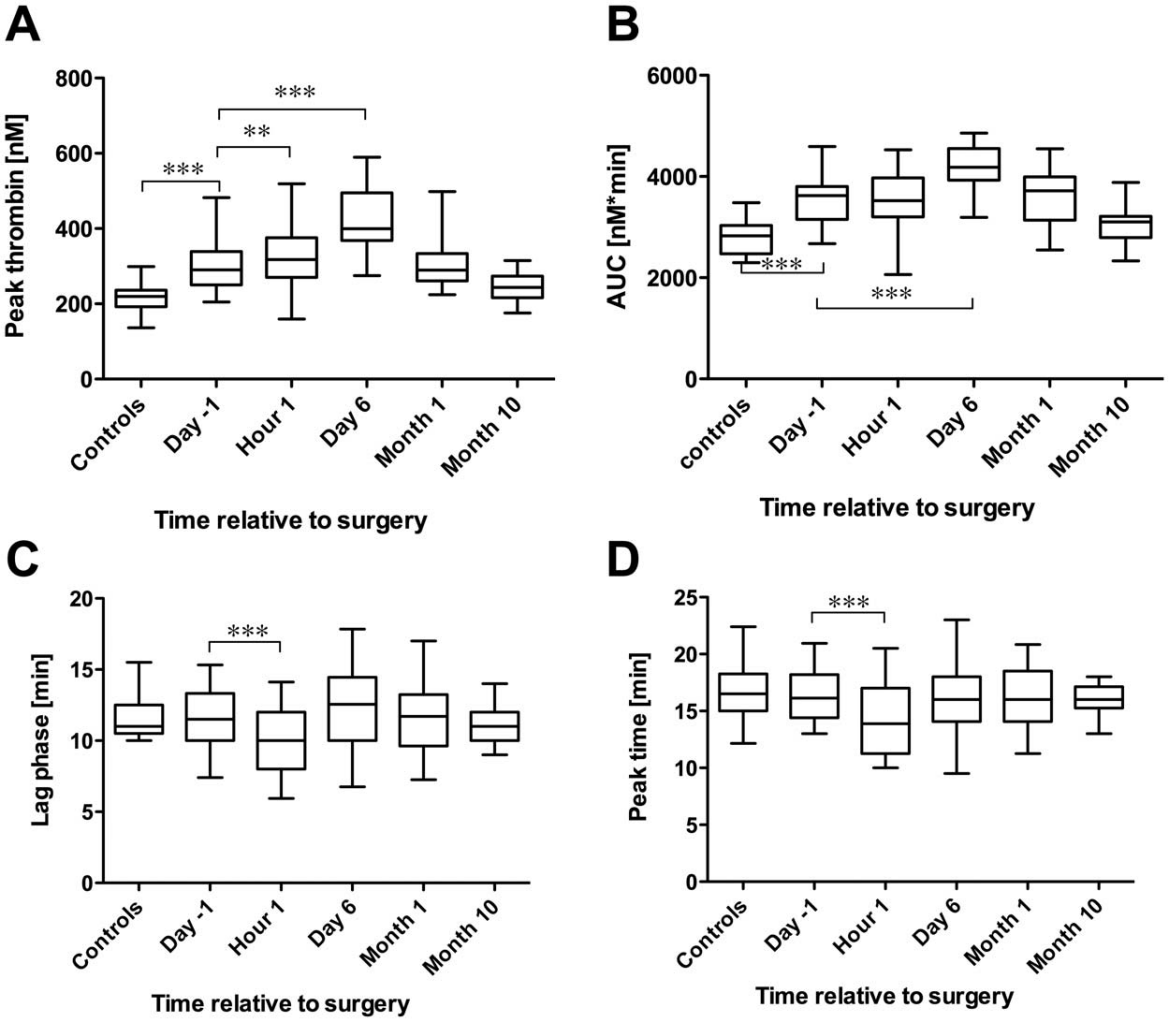
Thrombin generation parameters: Whiskers: 5–95 percentile graphs of peak thrombin (A), area under the curve (B), lag time (C) and peak time (D). Labels of significance: *p<0.05, **p<0.01, ***p<0.001.

Decreased antithrombin (AT) levels may increase the thrombin generation thus we assessed its changes in the study samples. Compared to the baseline, AT-levels were reduced one hour postoperatively, which however was not significant. As patients had been under prophylactic LMWH treatment and the presence of LMWH in plasma is expected to decrease peak thrombin level and AUC, the inhibitory effect of LMWH on FXa (aFXa activitiy) in the plasma samples was measured and found to be below the limit of detection in each of the samples (Table 2.).

The patients’ data was analyzed retrospectively for correlation between different clinical parameters and peak thrombin levels. Neither pathological tumor stage (pT) nor additional lymphadenectomy did alter TGA results significantly. Longer narcosis resulted in increased peak thrombin levels in the first postoperative sample (p = 0.024, unpaired t test with Welch’s correction). Baseline plasma peak thrombin levels of patients with elevated body mass index (BMI>25) were similar to the normal BMI patients, however a significant difference was found on the sixth postoperative day (p = 0.011, Mann Whitney test).

## Discussion

The aim of the present study was to evaluate the changes in hypercoagulable state after radical prostatectomy using thrombin generation assay. This measurement provides new metrics of hemorrhage control and vascular occlusion, and allows sufficient evaluation of an individual’s hemostatic competence and response to anticoagulant therapy [11], [12].

The results of our thrombin generation tests showed remarkable changes following radical prostatectomy. An even more significant difference in thrombin generation was found between the preoperative patient group and healthy control group. When compared to the controls, baseline thrombin generation and AUC were higher in the cancer patients which finding could be due to a variety of causes such as the presence of tumor cells, of microparticles and tissue factor [13]. Tissue factor and microparticles highly enhance procoagulant activity and thus influence the thrombin generation parameters [14], [15]. The difference of the TGA parameters between the controls and the preoperative values of the study population revealed increased procoagulant activity, which were further stimulated by the surgical intervention. The effect of the operation disappeared to the end of the first postoperative month. This supports the findings of other studies that the high-risk period after radical pelvic surgery ends before the first month [3]. The difference compared to the control group disappeared only by the 10 month check. The elevated baseline TG levels indicate the possible malignant procogulant activity of the prostate cancer, which is amplified by the surgical intervention.

Butenas et al confirmed that prothrombin and AT levels are dominant factors that influence thrombin generation when a synthetic “plasma” system was supplemented with factors II, X, XI, IX, VII, VIII and V, proteins C and S, AT and tissue factor pathway inhibitor [16]. A 50% decrease of AT increased thrombin production from 104% to 196%. However, as the decrease of AT in our samples was less than 10% and all levels remained within the reference range (80–120%), this cannot explain the remarkable increase in the thrombin generation seen in our study.

Changes in white blood cell counts reveal the acute immunological reaction to the tissue trauma, with an early compensation. Platelet count and conventional clotting tests varied somewhat, but all remained within the reference ranges, similar to what was observed after transurethral resection of benign prostatic hyperplasia [17]. Possible LMWH inhibitory actions on procoagulant factors can be excluded as at the moment of blood sampling, the levels in all samples were below the detection limit when measuring inhibition of FXa, consequently LMWH could not have influenced the result by decreasing procoagulant activity.

According to the correlation analysis of the FNG levels, conventional clotting times and TGA parameters we found that increased procoagulant activity is more readily detected when using the TGA, than by measuring changes in PT, APTT and TT levels. Elevated basic d dimer level was similar to published results [18], [19]. Fibrinogen-levels did show a slower response to tissue trauma, than d dimer and peak thrombin. Since we did not find a correlation between fibrinogen and peak thrombin generation, although fibrinogen influences thrombin generation [16], we conclude that in this altered balance of hemostasis as seen in the patients, it has a lesser contributing effect to the rise in peak thrombin levels or other TGA parameters.

Patients’ data were further analyzed for correlation between different clinical parameters and peak thrombin levels. Pathological tumor stage (pT) or additional lymphadenectomy was not significantly linked to TGA results. However, longer narcosis was observed to correlate with increased peak thrombin levels in the first postoperative sample. Baseline peak thrombin levels in plasma of patients with elevated body mass index (BMI>25) were similar to the normal BMI patients, but here a significant difference was seen on the sixth postoperative day. Beijers et al investigated the association between body composition and thrombin generation in plasma of 586 individuals and found an association with higher levels of thrombin generation in elderly women but not in men [20]. We hypothesise that body shape (characterized by BMI) may have a latent effect on procoagulant activity as BMI level caused difference only on the 6^th^ postoperative day but this statement needs further evaluation.

Limitations of our study are (i) the relatively low number of cases with as a consequence that the probability of having clinical signs of thrombotic events during the follow-up was limited; (ii) two of the patients were administered irradiation and hormone therapy fro the 6^th^ postoperative week; (iii) not all the data series showed Gaussian distribution, which decreased the statistical power; and (iv) there are other specific laboratory tests evaluating procoagulant activity, which were not evaluated in our study.

In conclusion, our study contributed to the knowledge of the pathophysiology of the hypercoagulable state of prostate cancer patients undergoing major pelvic surgery. Recent studies provided evidence that measurement of thrombin generation identifies patients at risk of venous thromboembolism [10], [6], It’s well known fact that surgical therapy of pelvic malignancies mean an additional risk of venous thromboembolism due to the nature of the intervention and the disease, but the stage of hypercoagulability during the postoperative period of radical prostatectomy has not been demonstrated yet. The goal of our present study was to evaluate this level with the use of a test which usefulness was proven by other studies. Although our case number was low to reach statistical probability for clinical thrombotic events, our results and recent articles present the power of thrombin generation test to detect such a high increase of the hypercoagulability, which indicate the risk of thrombotic events as shown in large studies [10].

Factors influencing thrombotic risk are still not well defined but our results suggest that increased narcosis and BMI may contribute to procoagulant activity in the postoperative period, but this statement needs further assessment.

Our study together with recently published papers assessing risk factors for arterial or venous thromboembolism suggests the need for individual anticoagulant and antiplatelet management plan likely to achieve a low incidence of thrombosis and prevent bleeding. [21]. To reach this goal, multidisciplinary approach is desirable.

Further evaluation of the hypercoagulable state in the postoperative period would lead urologists to an international and well supported consensus where e.g. anticoagulant treatment is considered for the first month only in order to provide a better clinical outcome.
